# Postural Control and Psychophysical State Following of Flight Simulator Session in Novice Pilots

**DOI:** 10.3389/fpubh.2022.788612

**Published:** 2022-02-03

**Authors:** Ewa Polak, Remigiusz Ślugaj, Adrianna Gardzińska

**Affiliations:** ^1^Academic Sports Centre, Rzeszow University of Technology, Rzeszow, Poland; ^2^Aviation Training Centre, Rzeszow University of Technology, Rzeszow, Poland

**Keywords:** flight simulator exposure, General Aviation, simulator sickness, postural sway, Simulator Sickness Questionnaire

## Abstract

Flight simulators can cause side effects usually called simulator sickness. Scientific research proves that postural instability can be an indicator of the occurrence of simulator sickness symptoms. This study aims to assess changes of postural control and psychophysical state in novice pilots following 2-h exposure to simulator conditions. The postural sway was quantified based on variables describing the displacement of the Center of Pressure (COP) generated in a quiet stance with eyes open (EO) and closed (EC). The psychophysical state was assessed using the Simulator Sickness Questionnaire (SSQ). The research was carried out in a group of 24 novice pilots who performed procedural and emergency flight exercises in the simulator at Instrument Meteorological Conditions. Each subject was examined twice: immediately before the simulator session (pre-exposure test), and just after the session (post-exposure test). The differences in postural stability between pre- and post-exposure to simulator conditions were assessed based on the normalized Romberg quotients, calculated for individual variables. The lower median values of all Romberg quotients confirmed the decreasing difference between the measures with eyes open and with eyes closed in the post-exposure tests. After the flight simulator session in both measurements (EO and EC) the values of the length of sway path (SP), the mean amplitude (MA), the sway area (SA) have changed. The visual contribution to postural sway control was reduced. The median values for all SSQ scores (total, nausea, oculomotor, and disorientation scales) were significantly higher in post-exposure tests. The largest increase was noted in the oculomotor SSQ scores (from 7.6 ± 7.6 to 37.9 ± 26.5). Over 50% of pilots participating in this study expressed symptoms typical of simulator sickness connected with visual induction: fatigue, eyestrain, difficulty focusing and difficulty concentrating. The severity of oculomotor and disorientation symptoms were rated as moderate (total SSQ score of more than 25 and <60). This study concludes that changes noted in the postural control and psychophysical state of the studied pilots after exposure to the flight simulator confirm the occurrence of the simulator sickness symptoms. Although, we did not find significant correlation of postural stability with SSQ scores.

## Introduction

Simulator flights are an important part of pilot training, regardless of the type of aviation and aircraft type. Modern flight simulators meet two main aviation objectives: to provide pilot training at the instructor's level, and at the student's level to learn to fly and to earn virtual flight hours that are useful for flying real aircraft and to simulate normal flight conditions, as well as adverse situations and spatial disorientation such as navigation instrument faults, power losses, loss of control of the aircraft, confusion illusion of references, illusion of the effect of black holes, among others, that would be dangerous and even catastrophic in a real flight (1). The use of simulators in aviation training, allows for consolidating habits, shaping the situational awareness of pilots, and increasing the effects of training aircraft crew. It also allows significantly reducing training costs and shortening learning time while ensuring pilots' and instructors' safety (2).

The evaluation, qualification and approval for flight simulators and flight simulation training devices (FSTD) must comply with the current regulatory standards, criteria, and requirements of aviation legislation, according to the required level of certification. The relevant criteria are primarily in the International Civil Aviation Organization (ICAO) documents, the European Union Aviation Safety Agency (EASA)/ Joint Aviation Requirements (JAR) regulation in Europe, and the Federal Acquisition Regulations (FAR) in the USA (3). The role and scope of simulator application increases in proportion to the development of aviation technology and equipment. The development of modern technologies and the improvement of the possibilities of virtual reality (VR) ensure that the training conditions in the modern simulators come closer to the situations that the pilot may encounter during the real flight. The pivot-and-swivel, cathode-ray tube (CRT) military flight simulators of the 1980s are being replaced by modern virtual reality and augmented reality systems with nearly unlimited potential for aviation training (4). The classical application of flight simulators in General Aviation is instrument training of pilots. Modern flight simulators are currently used for basic aviation learning, which consists of training for the Commercial Pilot License CPL(A), the Air Transport Pilot License ATPL(A) and the Instrument Rating (IR) courses. They are also mandatory devices for periodic pilot training e.g., Multi Crew Cooperation (MCC) or Crew Resources Management (CRM) and tests corresponding to the type or variant of the aircraft the pilot flies. Pilots joining the commercial airlines from flying schools, both civil and military, have to obligatory train on a flight simulator for specific types of aircraft. Only after obtaining the required experience on the simulator, the pilot can start real flights on a given type of aircraft (5).

The growing popularity and availability of flight simulators necessitate constant verification of existing knowledge about human reactions caused by exposure to their environments. The current scientific knowledge confirms that, the consequence of human exposure to the virtual environment (VE) may be specific side effects (6–9). Some of these effects are a natural adaptive response connected with the process of habituating, but others are classified as sickness signs and symptoms. A specific set of side effects that susceptible individuals may experience during and after exposure to flight simulator is usually referred to as a simulator sickness (10–12). It has been also referred to as simulator after effects or the simulator adaptation syndrome (13). Some scientists, as Ungs (14) have suggested that to limit the connotation with the disease, this phenomenon should be called simulator-induced syndrome, but this proposal did not find many supporters.

In simulator sickness research, other terms are used interchangeably, such as motion sickness (which arises from a susceptible individual's exposure to provocative motion), visually induced motion sickness (derived from visually provocative yet physically static environments) and cybersickness (induced by computer-generated displays or generally by virtual reality). The above-mentioned terms denote the types of syndromes, which may categorically and symptomatically overlap, but remain distinct from the simulator sickness symptoms (4, 15, 16). Havron and Butler, who first reported the simulator sickness in the 1950s, documented it as a set of specific reactions in the U. S. Navy helicopter flight trainers (17). Currently, this phenomenon is defined as motion sickness without true motion (4) or as a group of specific psychophysical ailments that may be experienced as a side effect during and after exposure to simulator or another VR environment - both static and dynamic (17–19).

The taxonomy of simulator sickness is more complex than that of motion sickness and includes symptoms typical of motion sickness, asthenopia (eyestrain), ataxia/vertigo (ataxia describes a lack of coordination while performing movements) (20). Apart from the feeling of general discomfort, the simulator's environment may cause such symptoms as fatigue, headaches, eyestrain, difficulty focusing, increased salivation, sweating, nausea, difficulty concentrating, head fullness, blurred vision (visual flashbacks), dizziness, vertigo, stomach awareness and burping (8, 17, 21). Kennedy et al. (22) clustered these sickness symptoms into three general types of effects or factors: nausea, oculomotor, and disorientation. Other physiological signs of sickness may include changes in cardiovascular, respiratory, gastrointestinal, biochemical, and temperature regulation functions. Postural and eye/hand incoordination are fewer known problems that may occur as a sole manifestation of sickness or may be present with other symptoms (16, 23). It is thought that any disruption of balance, coordination and motor control that results from exposure to a simulator may be a safety concern for pilots who need to walk, climb stairs, drive, or fly after a simulator training session (24). Some of these symptoms may persist or even worsen after leaving the simulator. Problems considered to be of greatest concern are the after-exposure effects such as illusory sensations of climbing and turning, perceived inversions of the visual field, and disturbed motor control expressed by postural instability, postural unsteadiness, or postural disequilibrium (23, 25, 26). Pilots who experience such sickness symptoms may be grounded following a simulated flight even for up to 24 h (8, 27).

To assess the simulator sickness both subjective and objective measures are used. Self-reported questionnaires are a tool used as a subjective measure (8, 12, 24). Among such tools the most widely used is Simulator Sickness Questionnaire (SSQ), developed and validated by Kennedy et al. (22). As objective tools, physiological measures are used. They are useful to describe changes in bodily cardiovascular, gastrointestinal, respiratory, biochemical, and temperature regulation functions during and after flight simulator exposure (25). Some researchers [e.g., (17, 28, 29)] have tested various physiological variables and some of them appear promising for evaluating simulator sickness without relying on self-reported measures or as a supportive method for questionnaires such as SSQ. As Kim et al. (30) have noted a solid combination of objective and subjective measures may offer a better solution for the evaluation of sickness symptoms.

Much research (17, 31–34) confirms that assessing the simulator sickness should consider the interaction of three components of the equilibrium system (visual, vestibular, and proprioceptive). Since the same components play a key role in the process of maintaining balance in a standing position, the postural instability or ataxia can indicate of the occurrence of simulator sickness symptoms. Postural instability can be measured using two types of floor-based tests: static (when subjects are asked to hold a given static posture) and dynamic (when subjects are asked to walk along a line) are usually used (8, 35). Of these, the static tests using time to stance during single leg (with eyes open or closed) or tandem stance (i.e., Sharpened Romberg), have given the most reliable results (35). Postural stability was also measured using motion analysis tools based on accelerometers (25, 35) or electromagnetic tracking system (15, 31, 33). Surprisingly, little research in this area has been conducted using a force or stabilometric platform which allows analysis of the center of pressure (COP) displacement (36). However, none of such tests has been thoroughly verified, and some studies have failed to show a correlation between the occurrence of simulator sickness symptoms and postural instability (4, 37, 38).

Since the 1950s, the simulator sickness was extensively studied, mainly for military training needs, and participant of these studies were U.S. Navy pilots [e.g., (11, 21, 39–41)]. The results of such studies and meta-analyses have indicated that the simulator sickness affected 10–88% or 12–60% of pilots tested on military simulators (12, 41). There is a lack of data on whether the simulator sickness is equally common in General Aviation. Research conducted in General Aviation pilots is still limited [e.g., (42)].

This study aims to assess changes of postural control and psychophysical state in novice pilots following exposure to simulator conditions. Other aim of this work is to determine the relationship between the results of post-exposure postural control and self-reported symptoms of adaptation to flight simulator conditions (called as simulator sickness symptoms). The authors hypothesized that postural sway changes can have the connections with a psychophysical state of pilots after exposure to a special kind of virtual reality, which is the fixed- based flight simulator.

## Materials and Methods

### Participants

The study participants were recruited from the students of the Rzeszów University of Technology. The students of second-degree studies in pilot specialization at Aeronautics and Space Technology were asked to join the study. They were informed about the purpose and course of this study, and they were asked to fill in the written form to accept or decline their participation. Voluntary consent was given by 27 men, who had participated in the last stage of integrated (theoretical and practical) training course for a “frozen” ATPL(A) that is for CPL(A) with ATPL(A) theory, Multi Engine Piston [MEP(L)] qualifications and, Multi Crew Cooperation (MCC). Graduates of such a course also obtain the qualifications to operate airplanes under instrument flight rules (IFR) and in instrumental meteorological conditions (IMC). To obtain full ATPL(A) qualifications, they still need to meet the requirements for flight experience−1,500 h flight time.

The inclusion criteria were as follow: male sex, active participation in the ATP course, valid aero-medical certificate, confirming no contraindications for performing independent flights, and declaration of good health on the day of the simulator exposure. Immediately before the flight session, three pilots reported feeling headache, fatigue, and difficulty concentrating. Due to not meeting the inclusion criteria, their results were rejected and for the final analysis, the results of 24 subjects were used.

Participation in the study was voluntary and its procedure was designed in accordance with the standards on personal data protection. All subjects were informed of the possibility of withdrawing from the study at any time. The study was approved by the local ethical committee and was done in accordance with the ethical standards specified by the Helsinki Declaration of 1975 as revised in 2013.

### Simulator Session

**Figure 1:**
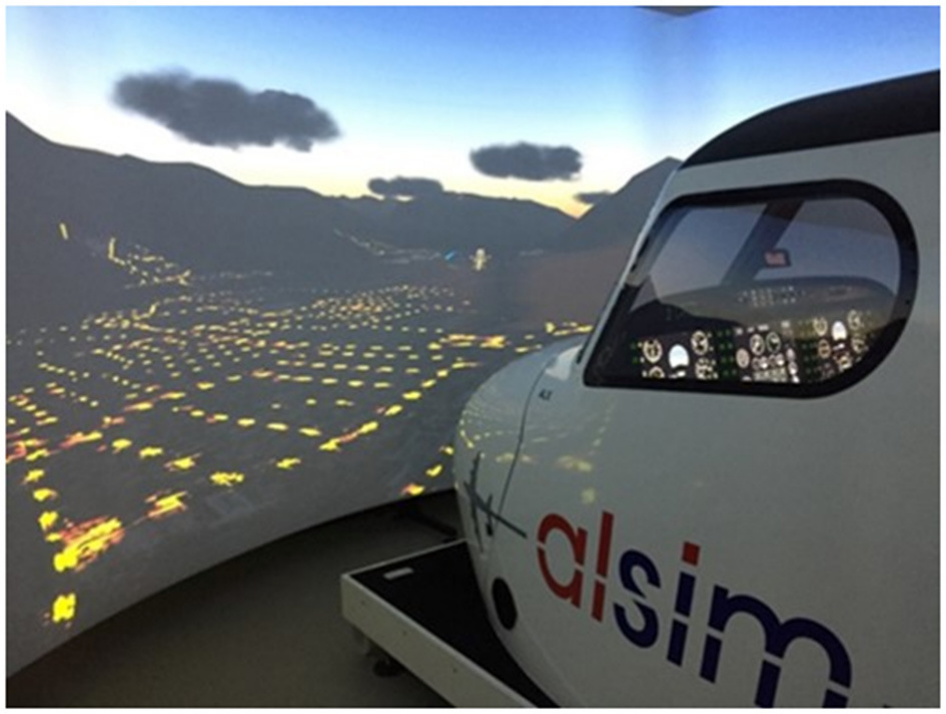
The Alsim ALX-30 flight simulator, made by Alsim Simulateurs, France (43).

The flight sessions during this study were performed according to the regular curriculum of integrated training courses for a “frozen” ATPL(A). All pilots participating in the study were maneuvered in the Alsim ALX-30 flight simulator (Figure 1).

This projection simulator is used for generic flight training, instrument training and is approved for MCC, CPL, ATPL, JOC (Jet Orientation Course) courses (3). It allows students to become familiar with the aircraft's systems. This device was certified as AATD according to U.S. Department of Transportation—Federal Aviation Administration (FAA) rules as well as CS-FSTD A—FNPT II and FNPT II MCC under EASA regulations. Alsim ALX-30 is a fixed- based training device with a fully functional and full-size replica of the two-seat cockpit with integral instructor cabin. Simulator cockpit is featured in all systems, their logics and complexity such as a multi-control panel including an instrument panel, full autopilot panel, GPS and LPV capabilities. This device allows for training the flight crew in selecting out of four different aircraft types and 10 different flight models to the extent that the on-board systems operate in a real plane. An integral part of the simulator is the High-Definition Visual System created by Alsim, which consists of a circular screen, three projectors and image distortion software. It provides the user a smooth, high-resolution 3D view, showing the spatial situation outside the cockpit with the 49 degrees vertical and 208 degrees horizontal field of view (43).

All subjects performed flight session lasting 2 h at the time specified in the ATP integrated training course schedule: between 8 a.m. and 8 p.m. During the session, the pilots performed the procedural flight exercises, training in take-off and approach to landing, flights in conditions of limited visibility (IMC), as well as exercises in emergency procedures. The detailed program of each flight session included exercises resulting from the course curriculum performed under the instructor's supervision.

### Measures and Data Collection

The data was collected over two-weeks at the Aviation Training Center of the Rzeszów University of Technology, Poland. Postural stability was quantified using data collected by static tests that characterized the postural sway during quiet bipedal stance. The Simulator Sickness Questionnaire (SSQ) scores quantified the psychophysical conditions. The measurements were conducted twice: immediately before the simulator session (pre-exposure tests), and just after the session (post-exposure tests). On the test day, each pilot filled out the pre-exposure SSQ and performed the pre-exposure postural stability test, then started the flight session in the simulator. Immediately after the session and exiting the simulator, pilot performed the post-exposure postural stability test and then he filled out the post-exposure SSQ. To ensure the minimum time between the pilot's exposure to the simulator conditions and the performance of postural stability tests the measures were carried out in the same room, where the simulator was located.

**Figure 2:**
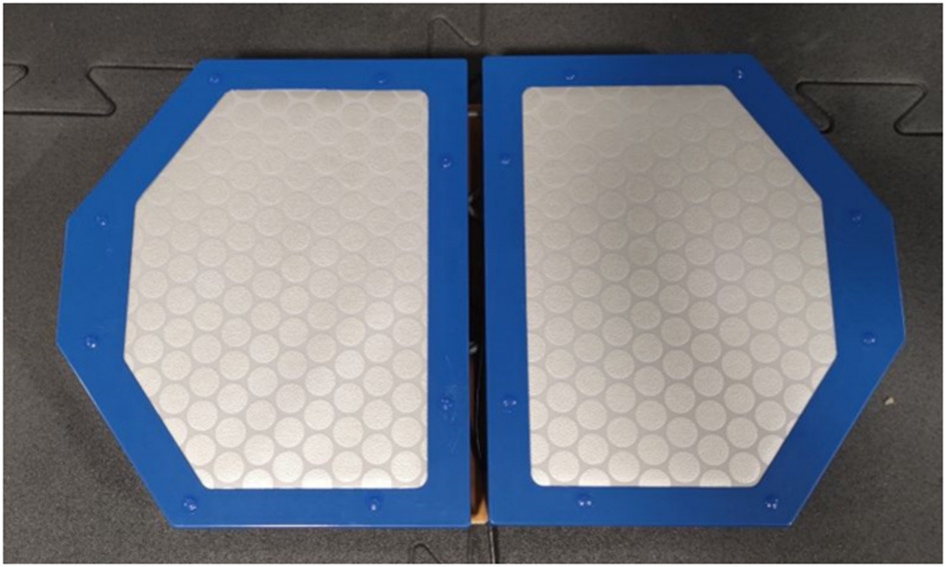
The CQ Stab 2P two-plates platform, made by CQ Elektronik, Poland (44).

The postural sway was quantified based on variables describing the displacement of the Center of Pressure (COP) generated on the platform in a quiet bipedal stance with eyes open and closed. Data was collected by the CQ Stab 2P two-plate platform (Figure 2).

The data registration was possible due to strain gauges placed in the corners of both platform plate. Signals from sensors were strengthened and transferred to analog-to-digital converter and were sent in a digital form to a control-communication module, responsible for converter data gathering and sending them to the computer. The results transformation was made by CQStab software. The applied static postural test was performed in accordance with the methodology recommended by the manufacturer of the CQ Stab 2P platform. Both plates of the platform were set in a parallel position and each test was preceded by a calibration of the platform. The test was conducted at the same environmental conditions for all subjects and consisted of two parts lasting 30 s with 30 s break. According to the study protocol, the subjects were in the upright position, bare-footed standing on the force platform with arms along the trunk, as recommended by the French Posturology Association—Rule 85 (45). The data was firstly recorded with the subject in the eyes open condition (EO) during 30 s and then with eyes closed (EC) for the same time duration. In the EO condition, the subjects were instructed to focus on central fixed target 1.5 m in front of the force platform. The COP was registered using the CQStab software as a point–dynamic parameter, changing its position in time (44).

The psychophysical state was assessed using the Simulator Sickness Questionnaire (SSQ). This questionnaire is a self-report symptom checklist, includes 16 symptoms associated with simulator sickness (17, 22). Though the SSQ has been primarily used for aviation purposes, studies connected with VR has been using this questionnaire extensively. It relied on indicating the level of severity for each of listed symptoms that pilots were experiencing before and after simulator session, using four levels of severity (none, slight, moderate, severe). The SSQ provides scores for three factors: Nausea (e.g., sweating, difficulty concentrating, and stomach awareness), Oculomotor Disturbance (e.g., headache, eyestrain, blurred vision) and Disorientation (e.g., head fullness, dizziness with open and closed eyes, vertigo). These factors are interdependent—some of the items are included in more than one factor, e.g., the score on difficulty focusing is using to assess the severity of oculomotor disturbance and disorientation. In total, there are five such items (38). The Total Score can be measured as well, and it is a composite created from the three subscales. It is the best single measure because it provides an index of the overall symptoms. All scores have as their lowest level a natural zero (no symptoms) and increase with increasing symptoms reported (17, 24, 38).

### Data Analysis

The data was analyzed in three following steps. The first step was analyses of variables recorded by CQStab software that described the COP displacement from the central point of stabilogram in two-dimensional coordinate system and represents a measure of sway stability with EO and EC. These variables were as follow: the length of sway path (SP), the mean amplitude (MA), the sway area (SA), and the mean frequency (MF). The differences between the values of the variables in the pre- and post-exposure postural tests were compared. The visual contribution to posture control was also analyzed by comparing the normalized Romberg quotients. They were calculated for all analyzed postural variables in pre- and post-exposure static postural tests, according to the formula used e.g., by Reed-Jones et al. (36):


(1) $$\begin{array}{r} RQ=\;\frac{EC\;score-EO\;score}{EC\;score+EO\;score}\;*\;100 \end{array}$$

This formula considers the total amount of body sway during both visual conditions (EO and EC). A Romberg quotient close to zero or negative indicates that the magnitude of body sway is similar or smaller in the condition with EC than with EO, i.e., visual information is less important for postural control (46).

The second step was analyses of pre- and post-exposure SSQ scores. It was done according to procedure of the interpretation recommended by Kennedy et al. (17). To calculate scores on each factor, all relevant items' scores were added (each factor consists of seven items) and multiplying the obtained sum by a specific weight. For nausea factor obtained sum was multiplied by 9.54 (therefore the scores on this scale range from 0 to 200.34), for disorientation by 13.92 (scores ranging from 0 to 292.32) and for oculomotor disturbance by 7.58 (with scores ranging from 0 to 159.18). A total score (TS) was derived by summing the raw (unscaled) three sub-factor scores and multiplied it by 3.74. Total scores can range from 0 to 235.62 (17, 38). The last step was the evaluation of the relationship between the results of the postural stability test and SSQ scores.

### Statistics

The descriptive statistics show the median value (*Me*), inter-quartile range (*IQR*), minimum values (*Min*) and maximum values (*Max*) in static postural test and SSQ scores as well. The *IQR* is a measure of statistical dispersion, which is the difference between the 75th and 25th percentiles of the data. For statistical analysis, nonparametric tests were used, because analyzed variables were not normally distributed. To assess variables distribution the Shapiro-Wilk test for normality was used. To determine the statistical significance of the differences between the results obtained in the postural stability tests from measures with EO and EC, as well as between the pre- and post-exposure results the Wilcoxon signed-ranks test was used. The same test was used to determine the significance of differences between pre- and post-exposure SSQ scores. The Spearman's rank correlation test allowed verifying the relationship between the results of postural stability tests and the psychophysical condition expressed by the SSQ results. All statistical analyses were carried out using the STATISTICA software from StatSoft Power Solutions, Inc., version 12. Differences for *p* ≤ 0.05 were considered statistically significant.

## Results

### Differences in Postural Stability Test Results

The novice pilots participating in the study were men with a mean age of 24.3 ± 1.5 years. They ranged in weight from 53.6 to 121.5 kg with a mean weight of 79.9 ± 15.9 kg and in height from 1.57 to 1.95 m with a mean height of 1.77 ± 0.8 m. Their aviation experience was as follow: time logged on planes ranged from 120 to 200 h with mean 156.2 ± 24.7 and time logged on simulator ranged from 10 to 50 h with mean 31.8 ± 13.7.

The results obtained during the pre-exposure postural stability tests are presented in Table 1. The descriptive statistics of these results are shown for both measures: with eyes open (EO) and with eyes closed (EC), conducted immediately before the flight simulator session.

**Table 1:** The descriptive statistics and differences in results of the pre-exposure postural stability tests with eyes open (EO) and eyes closed (EC).

**Variables**	**EO**		**EC**		**Tested value** ^**a**^	
	***Me ±IQR***	***Min-max***	***Me ±IQR***	***Min-max***	**Z**	***P-value***
SP pre (mm)	178.0 ± 37.0	120.0–235.0	238.5 ± 43.0	184.0–449.0	4.143	**<0.001**
MA pre (mm)	1.6 ± 1.5	1.1–5.3	3.0 ± 1.7	1.5–9.9	3.300	**<0.001**
SA pre (mm^2^)	85.0 ± 79.5	46.0–339.0	194.5 ± 120.0	92.0–730.0	3.771	**<0.001**
MF pre (Hz)	0.49 ± 0.31	0.17–0.87	0.44 ± 0.24	0.14–0.87	1.814	0.069

a *The Wilcoxon signed-ranks test, asymptotic significance (2-sided)*.

*SP Pre, pre-exposure length of sway path; MA Pre, pre-exposure mean amplitude; SA Pre, pre-exposure sway area; MF Pre, pre-exposure mean frequency; Me, median; IQR, inter-quartile range; Min-Max, minimum and maximum values*.

*Bolded p-values are statistically significant (α ≤ 0.05)*.

Data analysis indicates that the median values of SP, MA, and SA with EC were significantly greater than with EO. The results of measurements with EC indicate greater intra-group diversity, expressed by ranges of minimum and maximum values for above-mentioned variables. The value of the median of MF with EC was lower than with EO but this slightly difference was not statistically significant.

Table 2 presents the results obtained during the post-exposure postural stability tests, conducted immediately after the flight simulator session with EO and EC. Analysis of this data showed that the median values of SP, MA, and SA with EC were significantly greater than with EC. These variables also showed greater intra-group diversity with EC. It was noted that MF was characterized by an unchanged median value in both measures (EO and EC).

**Table 2:** The descriptive statistics and differences in results of the post-exposure postural stability tests with eyes open (EO) and eyes closed (EC).

**Variables**	**EO**		**EC**		**Tested value** ^**a**^	
	***Me ±IQR***	***Min-max***	***Me ±IQR***	***Min-max***	**Z**	***P-value***
SP post (mm)	169.5 ± 37.5	129.0–325.0	219.0 ± 46.0	155.0–408.0	4.171	**<0.001**
MA post (mm)	2.1 ± 1.0	0.8–4.0	2.8 ± 1.6	1.3–5.3	3.428	**<0.001**
SA post (mm^2^)	121.0 ± 60.5	32.0–320.0	201.0 ± 130.5	85.0–484.0	3.971	**<0.001**
MF post (Hz)	0.44 ± 0.18	0.23–0.91	0.44 ± 0.19	0.23–0.68	1.505	0.132

a *The Wilcoxon signed-ranks test, asymptotic significance (2-sided)*.

*SP Post, post-exposure length of sway path, MA Post, post-exposure mean amplitude, SA Post, post-exposure sway area, MF Post, post-exposure mean frequency, Me, median, IQR, inter-quartile range, Min-Max, minimum and maximum values*.

*Bolded p-values are statistically significant (α ≤ 0.05)*.

The analysis of differences between pre- and post-exposure variables showed that after 2-h flight simulator session in measures with EO the median of SP decreased and medians of MA and SA increased. It should be noted that the decrease in the median of SP was accompanied by an increase in the minimum and maximum values of this variable. In measures with EC median values of SP, MA decreased and SA increased. Such differences suggested that pilots' response to visual information changed in post-exposure postural tests. To assess the importance of visual information in postural control, normalized Romberg quotients were calculated for the length of sway path (RQSP), the mean amplitude (RQMA), the sway area (RQSA), and the mean frequency (RQMF).

**Figure 3:**
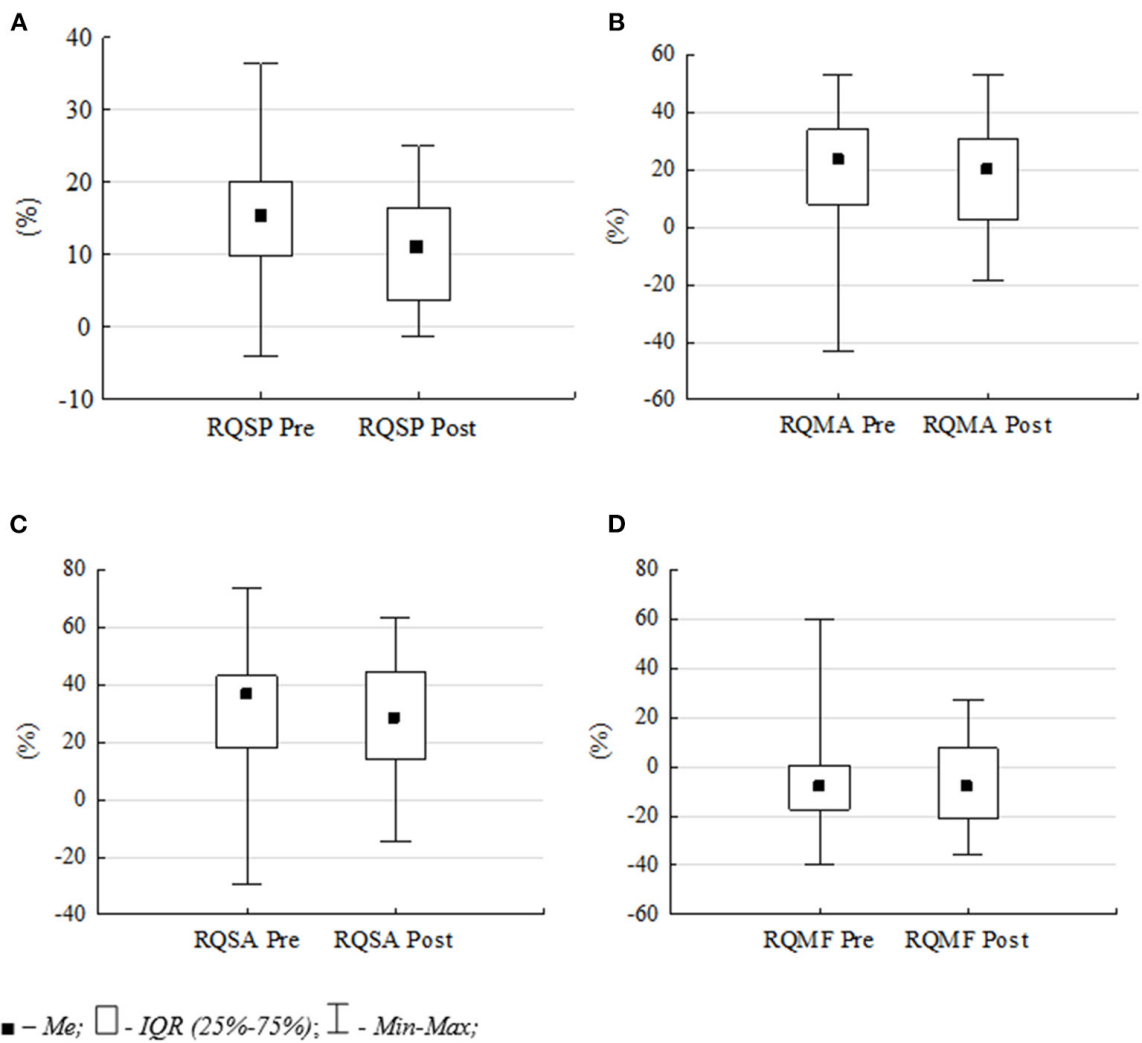
Differences in pre- and post-exposure normalized Romberg quotients: **(A)** RQSP, **(B)** RQMA, **(C)** RQSA and **(D)** RQMF. Data are median *Me*, inter-quartile range *IQR, Min* and *Max values*.

The comparison of the pre- and post-exposure normalized Romberg's quotients for all analyzed variables (Figure 3) allow verifying whether the 2-h flight session caused changes in pilots' postural control.

The comparison of normalized Romberg quotients confirms the decreasing difference between the measurements with EO and with EC in the post-exposure tests. The mean value of the RQSP decreased from 15.1 to 11.3%, the RQMA from 20.5 to 17.0% and the RQSA from 31.4 to 26.7%. This means that after flight simulator session, visual information was less important for postural control in subjects. The smallest changes were noted in the mean frequency of postural sway (MF). The mean value of the RQMF variable decreased from −5.7 to −6.1%. However, all these differences were not statistically significant.

### Differences in the SSQ Scores

**Figure 4:**
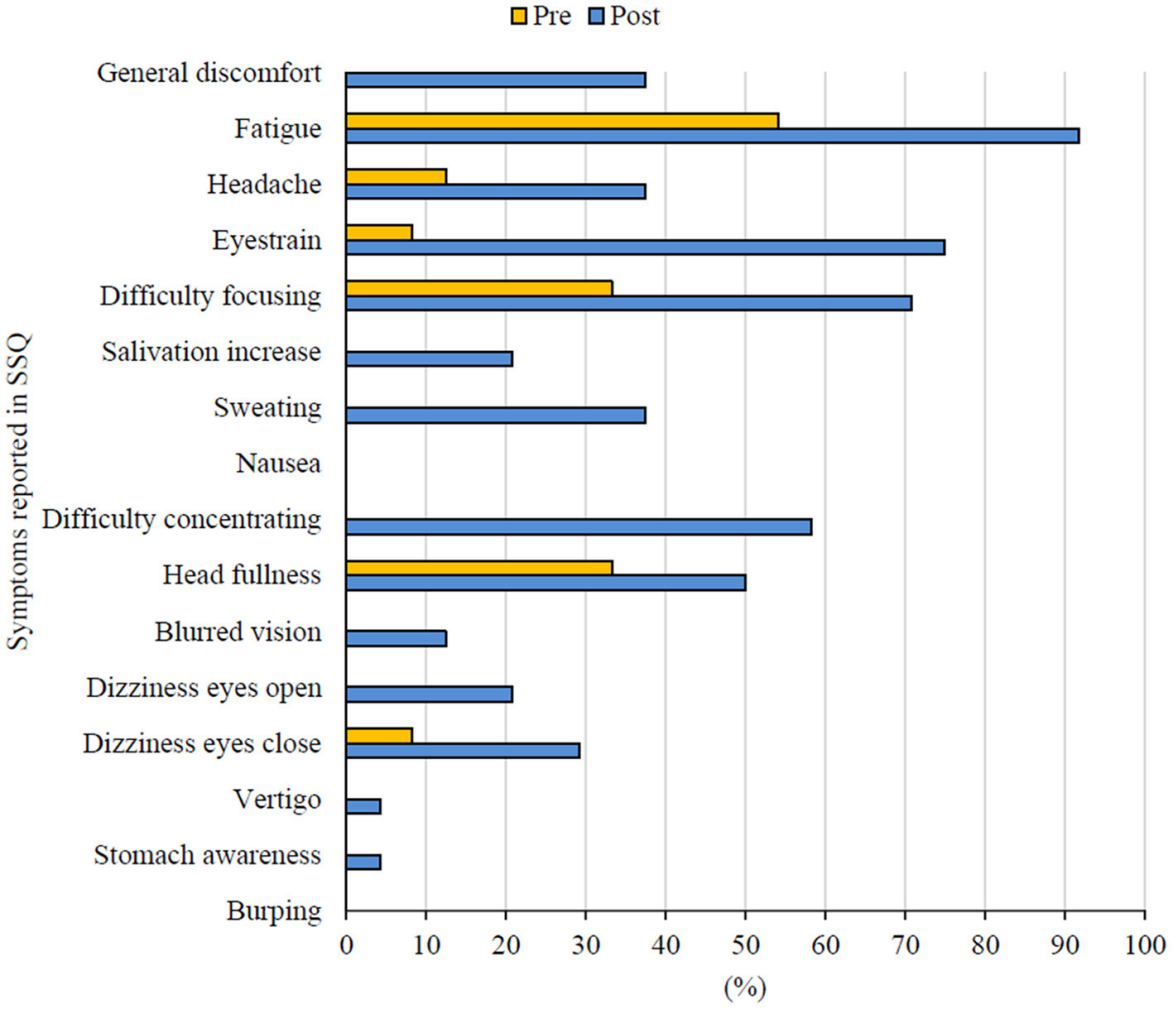
The frequency of symptoms reported in pre- and post-exposure SSQs.

The psychophysical state of the subjects was based on the analysis of the SSQ scores. This questionnaire investigated whether the 2-h flight simulator session caused the pilots to experience any symptoms considered typical for the simulator sickness. Figure 4 presents the percentage distribution of the frequency of reporting each of the 16 symptoms before and after exposure to the simulator conditions.

In pre-exposure SSQs, the most frequently reported experience was fatigue (54.2%), but 33% of the surveyed pilots also reported difficulty focusing and head fullness. In the post-exposure SSQ, the pilots reported experiencing a much larger number of symptoms (14 out of 16). Indeed, the tested pilots declared the severity of the reported symptoms as “slight” or “moderate”, but the frequency of the symptoms seems to be significant. More than half of the subjects declared a feeling of head fullness (50.0%), difficulty concentrating (58.3%), difficulty focusing (70.8%), eyestrain (75.0%) and fatigue (91.7%). None of the surveyed pilots reported any symptoms of nausea or burping.

Analysis of the SSQ data according to the methodology recommended by Kennedy et al. (23) made it possible to present numerical values for three subscales and the total score. Descriptive statistics of the pre- and post-exposure SSQ scores and the statistical significance of the observed differences are presented in Table 3.

**Table 3:** Descriptive statistics and differences in pre- and post-exposure SSQ scores.

**SSQ subscale**	**Pre**		**Post**		**Tested value** ^**a**^	
	***Me ±IQR***	***Min-max***	***Me ±IQR***	***Min-max***	***Z***	***P-value***
Nausea	0.0 ± 0.0	0.0–9.5	19.1 ± 19.1	0.0–38.2	3.723	**<0.001**
Oculomotor	7.6 ± 7.6	0.0–22.7	37.9 ± 26.5	7.6–68.2	4.286	**<0.001**
Disorientation	13.9 ± 13.9	0.0–41.7	27.8 ± 41.8	0.0–69.6	3.296	**<0.001**
Total score	7.5 ± 9.4	0.0–22.4	33.7 ± 22.4	3.7–56.1	4.286	**<** **0.001**

a *The Wilcoxon signed-ranks test, asymptotic significance (2-sided)*.

*Me, median; IQR, inter-quartile range; Min-Max, minimum and maximum values*.

*Bolded p-values are statistically significant (α ≤ 0.05)*.

The increase in the mean value of SSQ total score (from 7.5 ± 9.4 to 33.7 ± 22.4) confirms that effect of the exposure to the simulator conditions is the appearance of simulator sickness symptoms among pilots participating in this study. The mean values for all SSQ scores (total, nausea, oculomotor, and disorientation scales) were significantly higher in post-exposure tests. The largest increase was noted in the oculomotor SSQ scores (from 7.6 ± 7.6 to 37.9 ± 26.5).

### Relationships Between the Results of Post-exposure Postural Stability Test and SSQs Scores

The last step in the analysis was the verification of the relationship between the results of the postural stability test and the subjective assessment of the psychomotor state of the pilots. For this purpose, the correlation between the Romberg quotients for the analyzed variables and the SSQ scores was calculated.

Spearman's correlation coefficients (Table 4) took values close to zero in almost all correlations, so the strength of the relationship between the two variables was assessed as weak. Also, no statistically significant relationships were found.

**Table 4:** The Spearman's correlation coefficients in relations between the post-exposure Romberg's quotients and the post-exposure SSQ scores.

**Variable**	**SSQ N post**	**SSQ O post**	**SSQ D post**	**SSQ TS post**
RQSP post	0.155	0.278	0.149	0.261
RQMA post	−0.078	0.002	−0.011	−0.037
RQSA post	0.037	0.106	0.009	0.043
RQMF post	0.107	0.095	0.038	0.115

**Figure 5:**
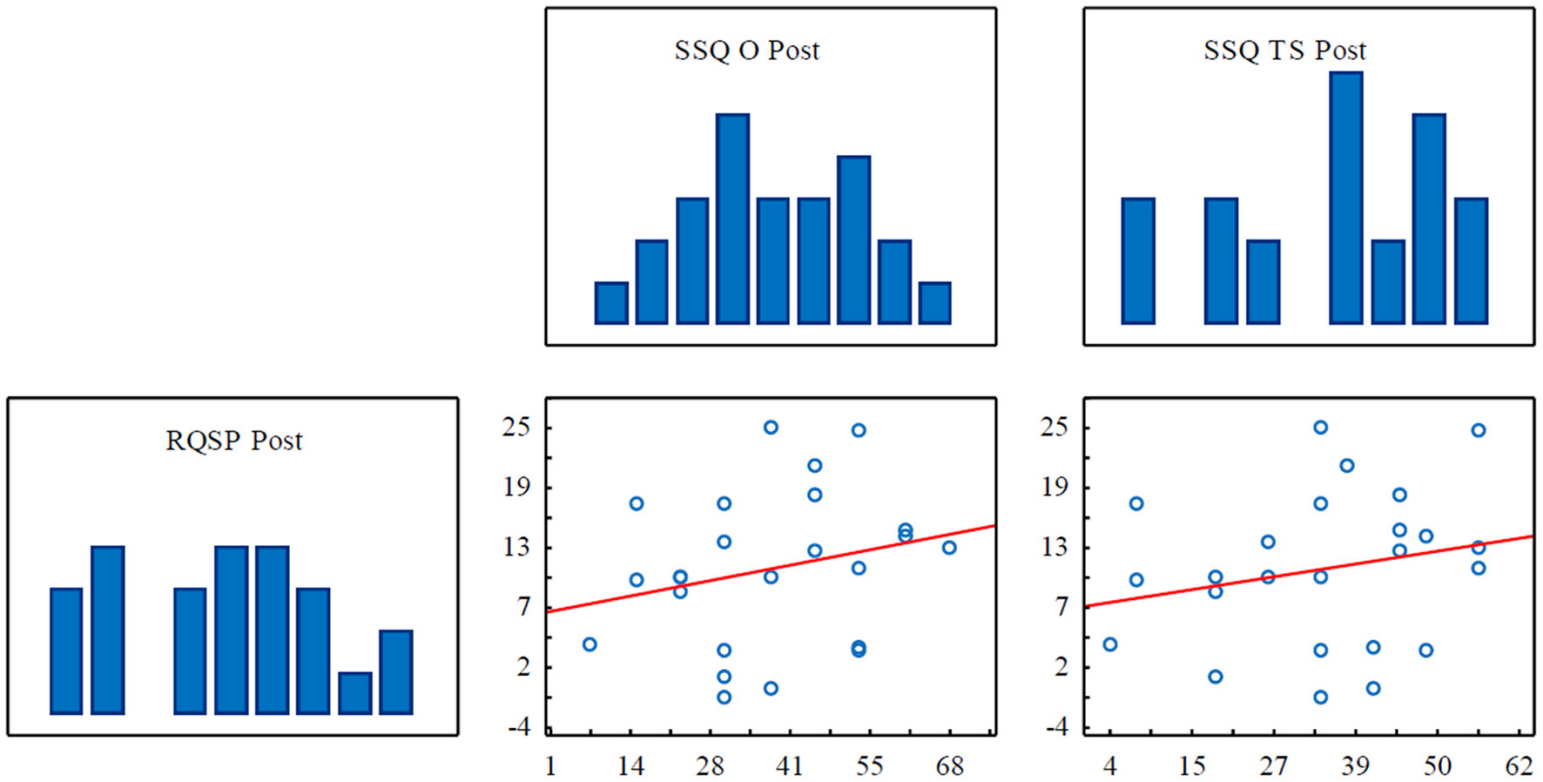
Scatterplots and histograms for Spearman's rank correlation showing the relationships between RQSP (Romberg quotients for sway path) and the SSQ oculomotor subscale scores as well as the SSQ total scores.

However, despite the lack of assumed relationships, it is worth paying attention to the relationship between the Romberg quotients for sway path and the SSQ scores. In two cases (Figure 5), the values of the correlation coefficients indicate a slightly stronger positive relationship. The first is the relationship between post-exposure RQSPs and the results of the post-exposure oculomotor SSQ scores, R_*s*_ = 0.278, p = 0.188. The second case is the relationship between post-exposure RQSPs and post-exposure total SSQ scores, R_*s*_ = 0.261, p = 0.217.

## Discussion

Past research has shown that the measure of the COP displacement by the force platform is a useful index of the assessing simulator sickness (25, 33, 47). It has been also widely proposed in biomechanical and clinical studies as an interesting feature among the above-mentioned ones that can provide good feedback from the user during immersion in a VR environment [e.g., (34, 48)]. Because it is one of the least constraining methods for a VR user compared to other physiological methods, we decided to use it in our study. Our main hypothesis was that postural control expressed by body sway variables will change in General Aviation novice pilots following of exposure to a special kind of VR facility - the fixed- based flight simulator Alism ALX-30.

Postural stability test performed on the force platform allowed us to obtain the main variables describing COP displacement during 30-second quiet bipedal standing with EO and EC. Analysis of the results indicate that the values of SP, MA, and SA with EC were significantly greater than with EO in both pre- and post-exposure postural stability tests. Such results would suggest that pilots have a visual preference in regulating postural stability (46). However, a comparison of the results of pre- and post-exposure tests showed that the exposure to simulator environment caused changes in postural variables in tested pilots.

To assess the visual contribution to postural sway control, the comparisons of normalized Romberg quotients were used. It was observed that after the simulator exposure difference between the results obtained with EO and with EC decreased. It indicates that the visual contribution to postural sway control in pilots was reduced as an adaptive response to the flight simulator environment. Probably greater vestibular and proprioceptive contribution on maintaining postural stability was caused by the fatigue of their sense of sight. The same reactions of the subjects were observed in study by Reed-Jones et al. (36), who analyzed the relationship between human adaptation symptoms and postural stability in a driving simulator. It is also worth noting that among the four analyzed Romberg quotients the biggest difference was noted in RQSA that decreased by 4.7%. Such result confirms observations of Takada et al. (47) who noticed that among various stabilometry parameters sway area of the COP displacement (SA) is the most useful feature for assessing Visually Inducted Motion Sickness (VIMS) symptoms. However, it should be noted that the analysis of the results in these studies did not show statistical significance, so the observed differences only show a trend that requires further research.

Much research conducted in the domain of simulator sickness confirmed that a widespread problem noted by trainees and subjects after exiting a flight simulator is ataxia, which is defined as postural instability, postural unsteadiness, or postural disequilibrium [e.g., (17, 26, 30)]. The reasons for this phenomenon have been described in previous studies. Postural control stabilizes the human body in space by integrating sensory input (somatosensory, visual, and vestibular) about body position with motor output to coordinate the action of muscles and keep the body's center of mass within its base of support. It relies on the control of two reflexes: the vestibulo-ocular reflex, which is responsible for stabilizing images on the retina, and the vestibulo-spinal reflex, which is responsible for maintaining body postural stability while in motion. If a conflict arises between the visual and vestibular sensory inputs, postural instability, and simulator sickness can occur (35). The most common theory explaining simulator sickness onset is the perceptual conflict model (the Neural Mismatch Model), which was created by Reason (49). The description of this theory can be as follows: the visual system perceives moving scenery, while the vestibular and proprioceptive cues suggest the subject is stationary. Immersion in VR environment causes a vestibulo-visual conflict, and as a result—a set of sickness symptoms (42). Such are the conditions in modern flight simulator with a great field of view, which presents an altered sensory environment. The simulator, by generating visual changes, forces the pilot to adapt to the new environment that occurs in the operator's visual and vestibular sensory systems. When leaving the simulator environment, pilot must adapt to natural conditions again. Upon return to the “normal” environment, balance and equilibrium may be disrupted until the pilot progresses through re-adaptation (48).

It should be noted that each person is not susceptible to simulator sickness in the same way (50). Kennedy and Stanney (25) found that sometimes post adaptation phenomena in the form of postural disruption can occur even when sickness is absent. Alternatively, postural instability may not be seen to a significant degree, but symptoms of sickness may be present. Therefore, just assessing postural control and analyzing the body sway may not be enough to indicate simulator sickness as some sickness symptoms may be hidden. Voluntary (corresponding to movements controlled by a person) vs. involuntary (corresponding to movements not controlled by the person, indicating potential sickness) movements are hardly distinguishable. As Chardonnet et al. (50) suggested the body sway can be an efficient feature to indicate the occurrence of sickness symptoms in a VR application but in conjunction with the Simulator Sickness Questionnaire (SSQ). Obtained in this study, SSQ scores allowed assessment of the frequency of symptoms reported by pilots associated with adaptation to flight simulator environment. The post-exposure SSQs scores indicate that more than 50% of the surveyed pilots experienced such sickness symptoms as: fatigue, eyestrain, difficulty focusing and difficulty concentrating. None of the subjects reported suffering from nausea or burping. These symptoms, experienced by the studied pilots, belong to the oculomotor scale. Therefore, analysis of subscales scores showed that the largest increase was noted in the oculomotor SSQ scores (from 7.6 ± 7.6 to 37.9 ± 26.5). Such results confirm the observations of other authors, such as Kennedy and Drexler (16), who proved that simulators tend to have disproportionately high oculomotor symptomatology (and low disorientation reports), while other VR environments tend to have high disorientation symptomatology (and moderate or low oculomotor reports). Analysis of total SSQ scores demonstrated the severity of the simulator sickness symptoms reported in studied pilots. The severity of nausea symptoms was rated as slight (total SSQ score of 25 or less). The severity of oculomotor and disorientation symptoms were rated as moderate (total SSQ score of more than 25 and <60). The analysis of changes in severity was not assessed. This study also hypothesized that postural sway changes could be related to psychophysical state of pilots after exposure to simulator environment. We were unable to confirm this relationship. Similar observation was noted in study by Cobb (35) who also did not find significant correlation of postural stability with SSQ scores.

It can therefore be concluded that symptoms typical of simulator sickness connected with visual induction were noted in pilots participating in this study. The cause of such symptoms experience can be explained by the Eye Movement Theory developed by Ebenholtz (51). According to this theory the vagus nerve stimulation is a main cause of simulator sickness. It is initiated by the optokinetic nystagmus and vestibular ocular response, which create tension in the eye muscles. In such condition the vagus nerve is stimulated, which can lead to unpleasant symptoms such as headaches, disorientation, and oculomotor effects (e. g., blurred vision) (38).

Nonetheless, some limitations of this study should be noted. Firstly, the size of the study group was limited by the number of students who participated in the aviation training carried out as part of their study program. Future research should consider a much larger group size. Secondly, pilots participating in this study had various levels of experience in flight simulators. It should be remembered that pilots with longer experience in operating the flight simulator could learn how to minimize the side effects caused by the simulator environment. Lastly, the tests did not include repeated measurements after exiting the simulator. It would also be advisable to contact the participants after the study and ask them if they experienced any unpleasant aftereffects of simulator exposure.

Until the technology reaches the point when the simulator sickness will be wholly preventable, some standards should be developed when it comes to research on simulator sickness. The issue of how often the simulator sickness symptoms should be measured (not only during the experimental trial, but also after it), should be addressed. Also important for future scientific study is to look for activities that can reduce the occurrence of simulator sickness. Past research has shown that repeated simulator exposures are an effective method to prevent simulator sickness and adapt to the VR environment (17, 52). It would be advisable to test the tendency of a new virtual reality tool to evoke the simulator sickness symptoms in the three above discussed dimensions: temporal pattern of the symptoms' progression, adaptation possibility and persistence of symptoms after exposure (50, 53). These parameters would provide vital information on how long the training should be, to provide the user with an enjoyable experience and to prevent unpleasant sensations. This issue appears to be exceptionally crucial for professional training simulators, where the quality of the experience may have an influence on results of the training session.

## Conclusion

This study concludes that changes noted in the postural control and psychophysical state of the studied pilots after exposure to the flight simulator confirm the occurrence of symptoms typical of simulator sickness. After the flight simulator session, the mean values of SP, MA, and SA with EO increased and with EC decreased. The visual contribution to postural sway control was reduced as an adaptive response to the flight simulator environment. The mean values for all SSQ scores (total, nausea, oculomotor, and disorientation scales) were significantly higher in post-exposure tests. The largest increase was noted in the oculomotor SSQ scores (from 7.6 ± 7.6 to 37.9 ± 26.5). Over 50% of pilots participating in this study expressed symptoms typical of simulator sickness connected with visual induction: fatigue, eyestrain, difficulty focusing and difficulty concentrating. The severity of oculomotor and disorientation symptoms were rated as moderate (total SSQ score of more than 25 and <60). We did not find significant correlation of postural stability with SSQ scores.

We considered that monitoring of the body postural sway during upright standing expressed by the Romberg quotients in conjunction with SSQs would be an efficient feature to indicate the prediction of simulator sickness in pilots. It can be a useful feature for the assessment of the human reaction on the exposition of the flight simulator as well as the other VR environments. Observation and analysis of psychophysical reactions of the simulator operator makes it possible to introduce additional actions to the content of aviation training to improve individual adaptation abilities for pilots who are susceptible to simulator sickness symptoms.

## Data Availability Statement

The datasets presented in this article are not readily available because of protection of personal data and information on the health status of study participants. Requests to access the datasets should be directed to Ewa Polak, e.polak@prz.edu.pl.

## Ethics Statement

The studies involving human participants were reviewed and approved by Bioethics Committee at Rzeszow University, Poland. The patients/participants provided their written informed consent to participate in this study.

## Author Contributions

EP conceived and designed the study, statistically analyzed, interpreted the data, and wrote the manuscript. RŚ recruited the subjects, made the measures, and interpreted the data. AG conceived and designed the study, interpreted the data, and wrote the manuscript. All authors listed have made a substantial, direct, intellectual contribution to the work approved it for publication, read, and approved the definitive version of the manuscript.

## Funding

This study was conducted as a part of the scientific project of Rzeszów University of Technology (DS.DL.19.001).

## Conflict of Interest

The authors declare that the research was conducted in the absence of any commercial or financial relationships that could be construed as a potential conflict of interest.

## Publisher's Note

All claims expressed in this article are solely those of the authors and do not necessarily represent those of their affiliated organizations, or those of the publisher, the editors and the reviewers. Any product that may be evaluated in this article, or claim that may be made by its manufacturer, is not guaranteed or endorsed by the publisher.
